# Supplementary material for: Tracking the extensive three-dimensional motion of single ions by an engineered point-spread function
Source: Nat Commun. 2024 Aug 1;15:6483. doi: 10.1038/s41467-024-49701-3 (PMC11294470; doi:10.1038/s41467-024-49701-3)
Supplement: Supplementary file 1 — Supplementary Information [file 41467_2024_49701_MOESM1_ESM.pdf]

Supplementary information: Tracking the extensive three-dimensional motion of single ions by an engineered point spread function

Yong-zhuang Zhou,[1,*] Man-chao Zhang,[1,2,3,*] Wen-bo Su,[1,3] Chun-wang Wu,[1,3]
Yi Xie,[1,3] Ting Chen,[1,3] Wei Wu,[1,3,4] Ping-xing Chen,[1,3,4,†] and Jie Zhang[1,3,‡]

[1] *Institute for Quantum Science and Technology, College of Science, National University of Defense Technology, Changsha 410073, China*

[2] *Northwest Institute of Nuclear Technology, Xi'an 710024, China*

[3] *Hunan Key Laboratory of Mechanism and Technology of Quantum Information, Changsha 410073, China*

[4] *Hefei National Laboratory, Hefei 230088, China*

SUPPLEMENTARY NOTE 1. FORCED VIBRATION OF A SINGLE TRAPPED ION

As the ion is trapped in a harmonic potential, it experiences secular oscillations in three dimensions [1]. After Doppler cooling, the motional amplitude of the ion is just a few tens of nanometers. Therefore, to show the extensive tracking range of our method, one strategy is to generate forced vibration with larger motional scales by applying RF driving signals along three orthogonal directions, as shown in Supplementary Fig. 1. Due to the single helical coil resonator used in our system, the secular modes along x, y axes are almost degenerate, and the independent control along these axes cannot be achieved just by applying driving signals on the resonator. Alternatively, near the geometrical center of the trap, there are two pairs of additional electrodes 3.85 mm away from the ion for the radial micromotion compensation. This means we can apply two driving signals on these electrodes to generate radial forced vibrations, as shown in Supplementary Fig. 1(a). For the axial motion, however, one of the tips can be utilized. In the figure, U_h, U_v, U_{dc} represent the DC voltages applied on horizontal compensation electrodes, vertical compensation electrodes as well as one of the axial tips after ion loading and micromotion compensation respectively, while F_h, F_v, F_{dc} indicate the RF driving signals applied for forced vibrations along h, v, z directions. Generally, the DC voltages and RF signals should not be combined directly to avoid the damage on signal generators and noise introduction. Here, we design an additional circuit, as shown in Supplementary Fig. 1(b). There is a resistance with 100k Ohms and a high-voltage capacitor with 11 µF near the RF signal input to block DC voltages. Besides, a 4.7 mH inductor and a low pass filter [LPF, see Supplementary Fig. 1(c) for details] are employed on the DC voltage input side to reduce the influence of RF signal on the DC voltage source. It should be mentioned that, before connecting to electrodes, the composite signal is also purified by a low pass filter LPF1 with band DC-5 MHz, which is able to filter out higher frequency RF signals including the AC component induced from four blades at 32.27MHz.

Suppose the secular frequencies in both x and y directions are ν_r, and the axial mode is ν_z. When the RF driving signals in form of $F_i = V_i \cos(\omega_i t + \phi_i), i = h, v, z$ generated by an arbitrary wave generator are applied, the equations determining the 3-dimensional motion of the ion can be written as follows without considering micromotion

$$x''(t) + \frac{q\Omega^2}{2}\cos(\Omega t)x(t) - \frac{\sqrt{2}}{2}\frac{e}{m}\alpha_h F_h - \frac{\sqrt{2}}{2}\frac{e}{m}\alpha_v F_v = 0, \tag{1}$$

$$y''(t) - \frac{q\Omega^2}{2}\cos(\Omega t)y(t) + \frac{\sqrt{2}}{2}\frac{e}{m}\alpha_h F_h - \frac{\sqrt{2}}{2}\frac{e}{m}\alpha_v F_v = 0, \tag{2}$$

$$z''(t) + \nu_z^2 z(t) - \frac{e}{m}\alpha_z F_z = 0, \tag{3}$$

where $e = 1.6 \times 10^{-19}$ C and $m = 40 \times 1.67 \times 10^{-27}$ kg denote the charge and mass of the $^{40}Ca^+$ ion. $e\alpha_i F_i, i = h, v, z$ indicate the driving forces generated by RF signals on the ion, and for simplicity, we assume $\alpha_h = \alpha_v$. Ω is the frequency of trap RF driving signal, satisfying $\Omega \gg \nu_r$, and the coefficient q is expressed as $q = 2\sqrt{2}\nu_r/\Omega$. After laser cooling, the ion is nearly frozen in its equilibrium position, therefore the initial conditions to Eq. (1-3) are given by

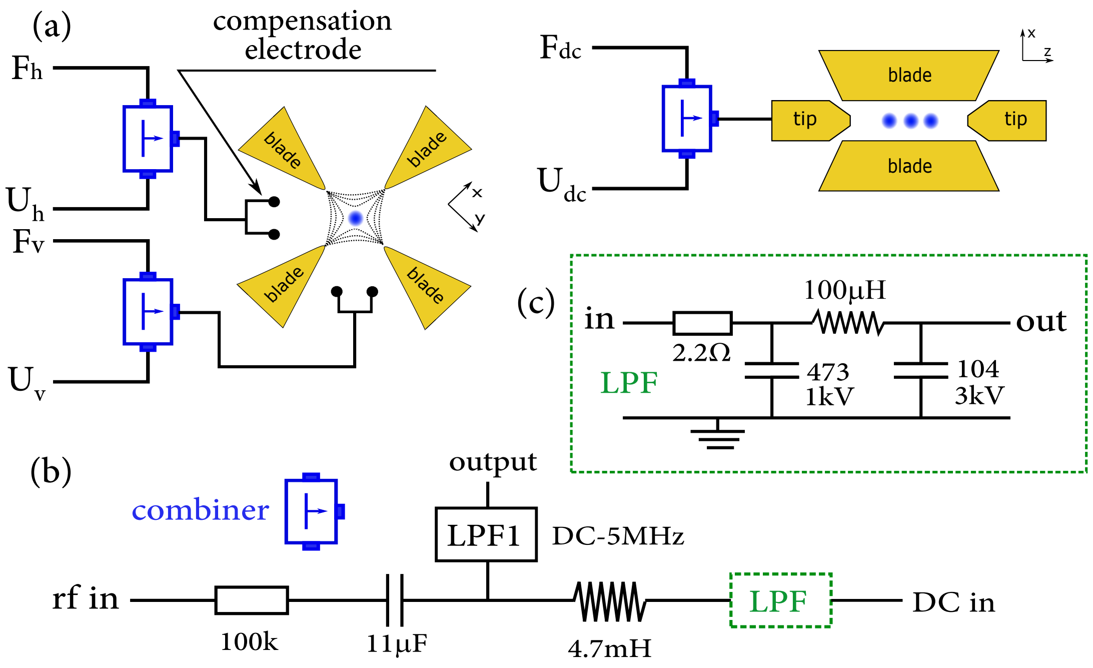

Supplementary Fig. 1. Scheme for 3-D forced vibration. (a) The radial driving signals are applied by connecting to the horizontal and vertical micromotion-compensation electrodes, while the signal along z axis is applied to one of the tips. U_h, U_v, U_{dc} represent the DC voltages for micromotion compensation and ion trapping, and F_h, F_v, F_dc are RF driving signals. (b) Added circuit for combining DC and RF driving voltages. (c) Design for a low-pass filter.

$i'(0) = i(0) = 0, i = x, y, z$. Following a law of transformation $h(t) = \frac{\sqrt{2}}{2}(x(t) - y(t)), v(t) = \frac{\sqrt{2}}{2}(x(t) + y(t))$, the analytical solutions can be approximately obtained in the case of $q \ll 1$:

$$h(t) = \frac{e\alpha_h V_h}{m} \frac{\sin\phi_h(\omega_h \sin\nu_r t - \nu_r \sin\omega_h t) - \nu_r \cos\phi_h(\cos\nu_r t - \cos\omega_h t)}{\nu_r^3 - \nu_r \omega_h^2}, \tag{4}$$

$$v(t) = \frac{e\alpha_v V_v}{m} \frac{\sin\phi_v(\omega_v \sin\nu_r t - \nu_r \sin\omega_v t) - \nu_r \cos\phi_v(\cos\nu_r t - \cos\omega_v t)}{\nu_r^3 - \nu_r \omega_v^2}, \tag{5}$$

$$z(t) = \frac{e\alpha_z V_z}{m} \frac{\sin\phi_z(\omega_z \sin\nu_z t - \nu_z \sin\omega_z t) - \nu_z \cos\phi_z(\cos\nu_z t - \cos\omega_z t)}{\nu_z^3 - \nu_z \omega_z^2}. \tag{6}$$

We can rewrite the above equations in a uniform expression by defining $\nu_h = \nu_v = \nu_r$

$$i(t) = \frac{e\alpha_i V_i}{m} \frac{\sin\phi_i(\omega_i \sin\nu_i t - \nu_i \sin\omega_i t) - \nu_i \cos\phi_i(\cos\nu_i t - \cos\omega_i t)}{\nu_i^3 - \nu_i \omega_i^2}, \quad i = h, v, z. \tag{7}$$

It indicates that the motion scale of the ion is proportional to V_i, and different initial phase ϕ_i may lead to quite different forced vibration, as shown in Supplementary Fig. 2. Compared to Supplementary Fig. 2(a) ($\phi_i = 0$), it is clear that a vibration with smaller modulation can be obtained in Supplementary Fig. 2(b) where $\phi_i = \pi/2$. Here, we propose an optimized shape of electrical pulse where an ion-shift RF signal is applied just before the driving signal with $\phi_i = 0$ [see Supplementary Fig. 3(a)]. In order to suppress the modulation induced by secular motion, the frequency of this extra ion-shift RF signal should be as small as possible, so that the ion can be shifted to its maximum displacement approximately adiabatically. Due to this ion-shift process, the initial conditions of Eq. (1-3) become $x(0) = \frac{e}{\sqrt{2}m}\alpha_h V_h/\nu_r^2 + \frac{e}{\sqrt{2}m}\alpha_v V_v/\nu_r^2$, $y(0) = -\frac{e}{\sqrt{2}m}\alpha_h V_h/\nu_r^2 + \frac{e}{\sqrt{2}m}\alpha_v V_v/\nu_r^2$, $z(0) = \frac{e}{m}\alpha_z V_z/\nu_z^2$, which actually indicate the motional amplitudes of forced vibrations. Then we obtain

$$i(t) = \frac{e\alpha_i V_i}{m} \frac{\cos(\omega_i t) - (\frac{\omega_i}{\nu_i})^2 \cos(\nu_i t)}{\nu_i^2 - \omega_i^2}, \quad i = h, v, z. \tag{8}$$

If $\omega_i/\nu_i \ll 1$, these equations can be further simplified to be

$$i(t) = \frac{e\alpha_i V_i}{m\nu_i^2}\cos(\omega_i t), \quad i = h, v, z. \tag{9}$$

This equation indicate the harmonic oscillations when the shift signals as well as $F_i, i = h, v, z$ are implemented, and their motional amplitudes are proportional to the strength V_i of the driving signals. We numerically simulate the forced vibration induced by the designed electrical pulse shown in Supplementary Fig. 3(a), and the result is represented by the blue-solid curve in Supplementary Fig. 3(b). It is well consistent with the red-dashed curve expected by Eq. (9), where little modulation is revealed compared to Supplementary Fig. 2.

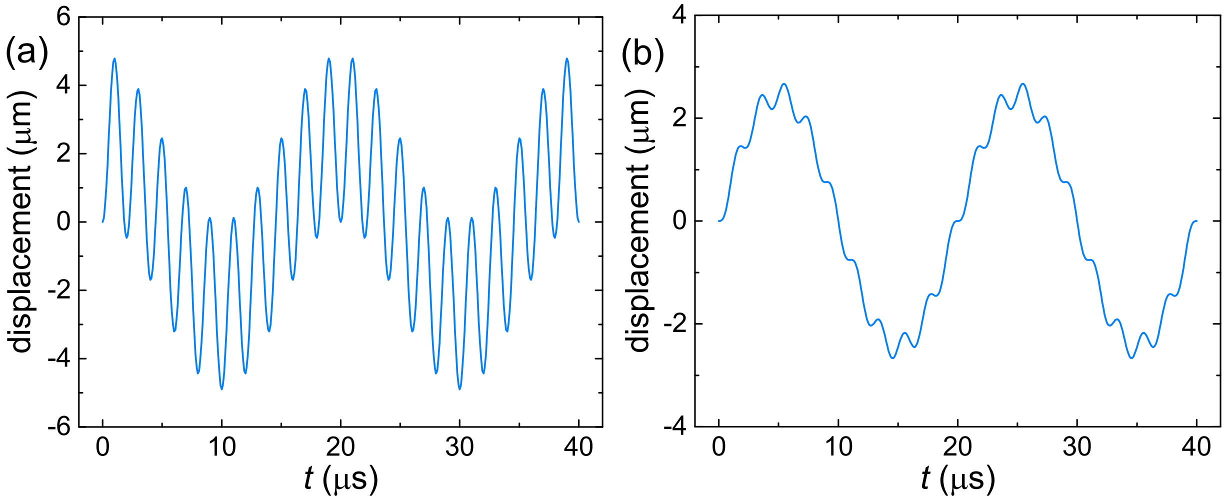

Supplementary Fig. 2. Forced vibration resulted from a sinusoidal signal with (a) $\phi_i = 0$ and (b) $\phi_i = \pi/2$. Other parameters used in the simulation are $V_i = 10$ V, $\alpha_i = 1$, ν_i =500 kHz, ω_i=50 kHz.

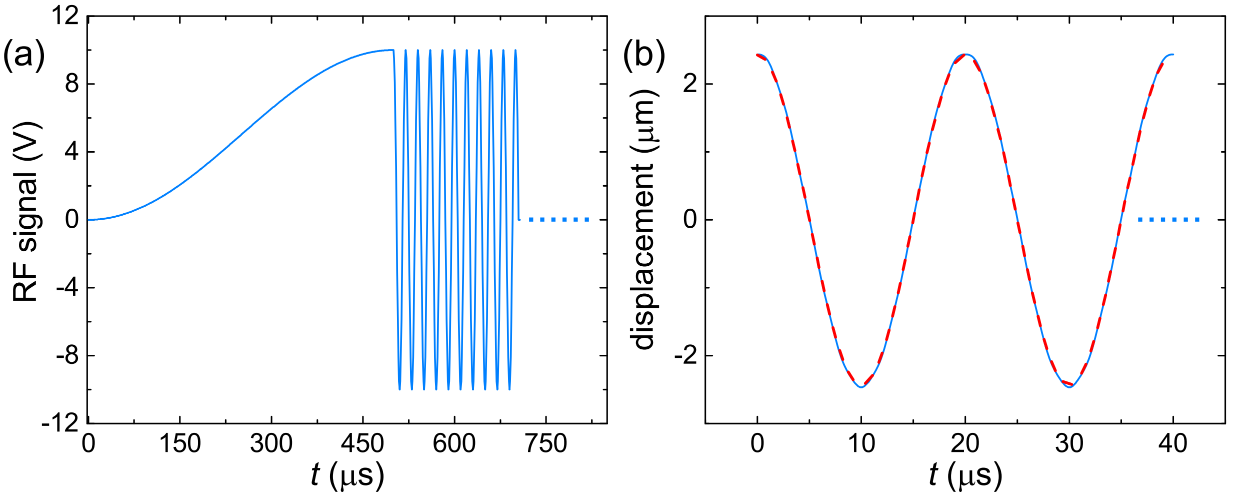

Supplementary Fig. 3. Forced vibration driven by a designed RF signal. (a) The designed signal is composed of two parts, the shift voltage at f_s=1 kHz and the driving RF signal. The function of shift voltage can be expressed as $V_i/2 - V_i/2\cos(f_s t)$, and the ion shift is accomplished within 500 µs. (b) The forced vibration driven by the designed RF signal shown in (a). $t = 0$ here corresponds to $t = 500$ µs in (a). The blue solid and red dashed curves represent the simulated result and the expected result derived from Eq. (9), respectively. Other parameters used in the simulation are $V_i = 10$ V, $\alpha_i = 1$, ν_i =500 kHz, ω_i=50 kHz.

SUPPLEMENTARY NOTE 2. IMAGING SEQUENCE FOR DATA ACQUISITION OF THREE-DIMENSIONAL MOTION

We use an electron-multiplying charge-coupled device (EMCCD) to collect the photons emitted from the trapped ions. The imaging area of this camera is 13.312×13.312 mm^2 with 1024×1024 pixels, therefore the size of each pixel is 13×13 µm^2. In our experiments, the driving frequency ω with period $\tau = 2\pi/\omega$ can be up to (2π)100 kHz. To trace the ion motion with decent precision, we sample at least 10 points within a motion period, corresponding to a detection duration $\Delta t < \tau/10$. Because the minimum exposure time of camera (10 µs) is larger than the sampling period; here, we propose an imaging sequence illustrated in Supplementary Fig. 4 to realize a data acquisition with higher temporal resolution and signal-to-noise ratio. The sequence is divided into 3 parts of setup, the RF driving signal, the 397-nm detection laser and the camera. When the driving signal is applied at $t = 0$, the camera is also triggered on by a high-level pulse. For various waiting time t_0 to observe the ion's motion at different oscillation phase, we turn on the 397-nm laser beam at $t = t_0 + n\tau, n = 0, 1, ..., N - 1$ for a duration of $\Delta t = 1$ µs. During these intervals, the ion can be excited from level $4^2S_{1/2}$ to $4^2P_{1/2}$, and then scatter fluorescence photons. This process takes place continually, which enables us to take images that reveal the positions of the ion. Note that due to the small Δt, the signal-to-noise ratio in a single detection interval is rather small. Therefore, in our scheme, the ion is driven for $N + 1/4$ periods with RF driving signal and accumulate all the photons collected during this process. Limited to the experimental conditions, we usually set several thousands of cycles to get enough fluorescence photons. Finally, the above sequence is cycled and all the obtained images are then accumulated to generate a single image for position analysis.

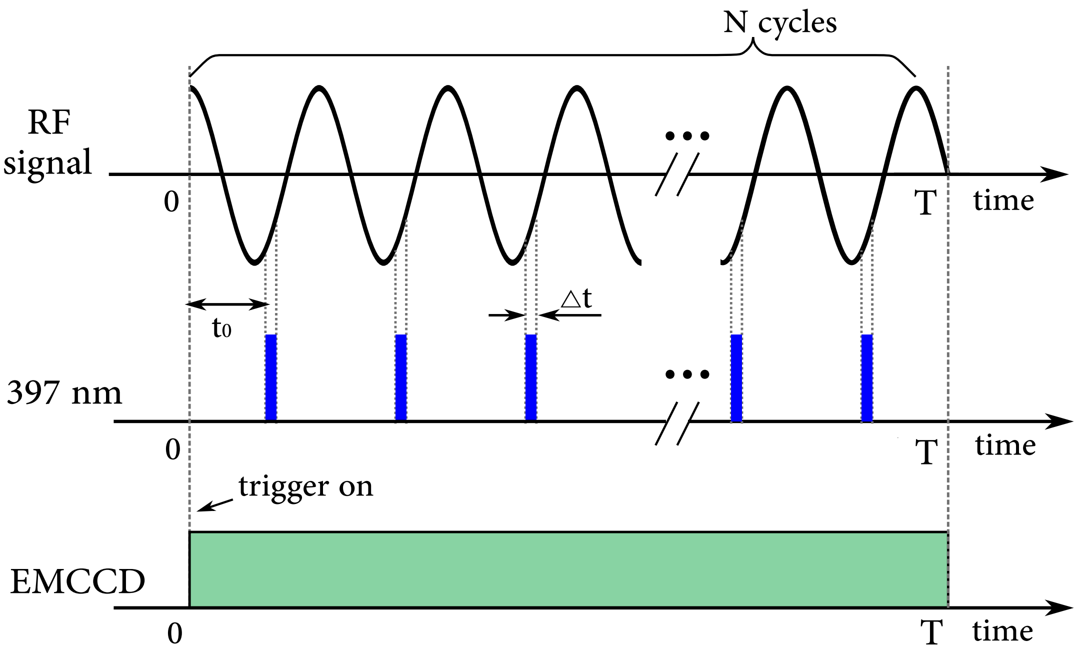

Supplementary Fig. 4. The imaging sequence for ion motion tracking. The fluorescence of the ion is collected by keeping the EMCCD on constantly, while applying the the 397 nm detection laser with fixed time delay and short pulse duration Δt =1 µs. Position for each fixed time delay is acquired by accumulating the photons in each iteration, and the full motion can be traced by varying the time delay till the whole period is covered.

SUPPLEMENTARY NOTE 3. ZIG (ZAG) MODE CONFIGURATION

Consider N identical ions with mass m and charge q trapped in a blade trap. When the ions are assumed to be strongly bounded in the radial directions but weakly confined along the axial direction, these ions will arrange themselves in a linear structure. Then the potential energy including harmonic trapping potential and ion-ion Coulomb interaction can be approximately written as

$$U = \frac{m}{2}\sum_{n=1}^{N}\nu_z^2 z_n^2 + \frac{q^2}{8\pi\epsilon_0}\sum_{n,m=1,n\neq m}^{N}\frac{1}{|z_n - z_m|}. \tag{10}$$

Here, we only take the axial potential into account, assuming a linear configuration with $x_n = y_n = 0$. The minimum-energy equilibrium structure $z_n^{(0)}$ can be determined by the following equation

$$\left[\frac{\partial U}{\partial z_n}\right]\Bigg|_{z_n = z_n^{(0)}} = 0. \tag{11}$$

It should be mentioned that for more than 3 ions, this equation can be solved numerically only. The distance between adjacent ions is minimal and increases as towards to the outside of the linear string.

The one-dimensional (1D) linear equilibrium configuration remains as long as the radial confinement is much stronger than the axial confinement. However, as the axial confinement is strengthened or equivalently the radial confinement is relaxed, a zig or zag structure will be generated. In this case, the conditions $x_n = y_n = 0$ does not hold anymore. If the position of the n-th ion is denoted as $\vec{r_n} = \{x_n, y_n, z_n\}$, then the 3D potential energy can be written as

$$U_{3D} = \frac{m}{2}\sum_{n=1}^{N}(\nu_x^2 x_n^2 + \nu_y^2 y_n^2 + \nu_z^2 z_n^2) + \frac{q^2}{8\pi\epsilon_0}\sum_{n,m=1,n\neq m}^{N}\frac{1}{|\vec{r_n} - \vec{r_m}|}, \tag{12}$$

where ν_x, ν_y represent the secular motion frequencies along x, y axes respectively. Here, we define two trap anisotropy parameters as $\alpha_x = \frac{\nu_x}{\nu_z}$ and $\alpha_y = \frac{\nu_y}{\nu_z}$. If the ions' positions are re-scaled as $\vec{r_n}/a \rightarrow \vec{r_n}'$ with $a = (q^2/4\pi\epsilon_0 m\nu_z^2)^{1/3}$, then Eq. (12) is simplfied to

$$U_{3D} = U_0\Big(\frac{1}{2}\sum_{n=1}^{N}(\alpha_x^2 x_n'^2 + \alpha_y^2 y_n'^2 + z_n'^2) + \frac{1}{2}\sum_{n,m=1,n\neq m}^{N}\frac{1}{|\vec{r'_n} - \vec{r'_m}|}\Big), \tag{13}$$

where $U_0 = m\nu_z^2 a^2$. Then the 3D structure of ion crystal can be numerically derived by minimizing the potential U_{3D}. Generally, the equilibrium structure mainly relies on α_x and α_y. Due to the slight asymmetry of trap, the radial secular motional modes are degenerated from each other. If we define $\nu_i = \min\{\nu_x, \nu_y\}$, as α_i decreases acrossing a threshold α_c, the linearly arranged ions will no longer have the minimum energy, and alternatively they will exhibit a zig or zag spatial configuration, which lies in a 2D plane expanded by axes z and i [2]. Note that both zig and zag structures have the same potential energy. Thus we may observe transitions between the two configurations due to the inevitable thermal fluctuations in experiments.

SUPPLEMENTARY NOTE 4. GENERATION AND IMPLEMENTATION OF THE HELICAL PHASE MASK

Engineered PSFs are typically achieved on microscopes equipped with a $4f$ relay system to access its Fourier plane (i.e. the back focal plane of the objective which likely lies within the objective's rear aperture [3]), the PSF in the image space is therefore expressed as the Fourier transform of the modulated pupil function [4, 5]

$$I_{\text{PSF}} \propto \left|\mathcal{F}\left\{A(\rho,\phi)\mathrm{e}^{2\pi i(\psi(\rho,\phi)+D(\rho,z))}\right\}\right|^2, \tag{14}$$

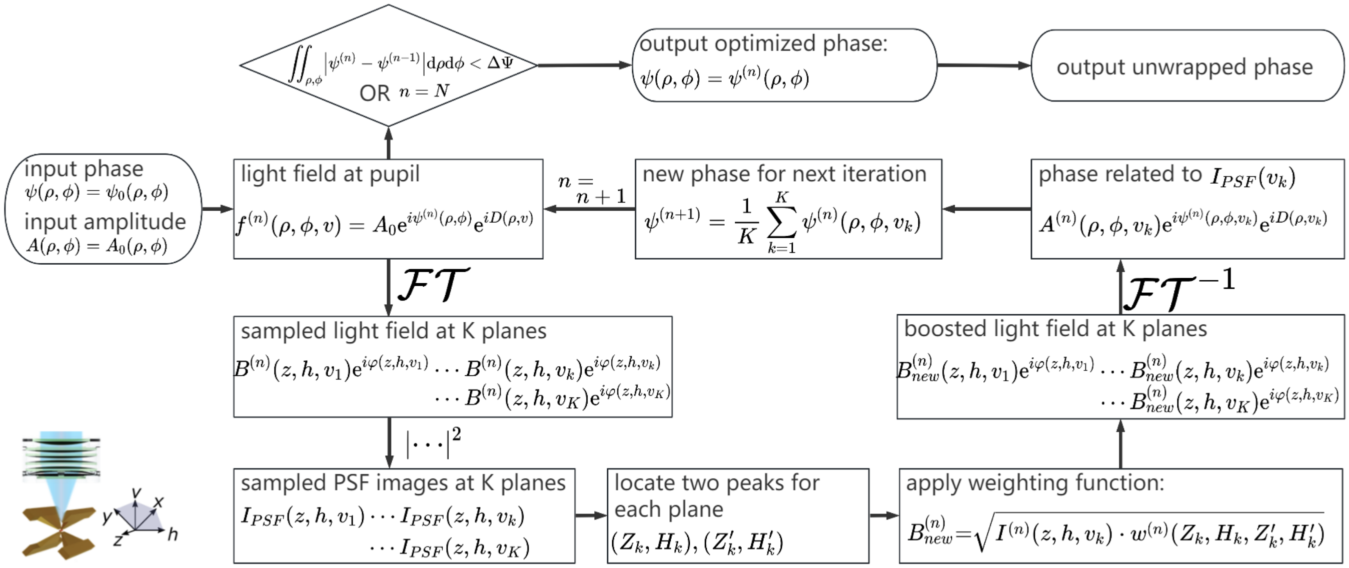

Supplementary Fig. 5. Iterative algorithm to generate the helical phase modulation. Input amplitude A_0 is a clear aperture, input phase ψ_0 is defined by Eq. (15). A and ϕ are the amplitude and phase at the Fourier plane; B and ϕ are amplitude and phase at the image space. K is the total number of sampling planes in the image space, $I_{PSF}(z, h, v_k)$ refers to the lateral intensity distribution of the PSF at $v = v_k$. w is the weighting function.

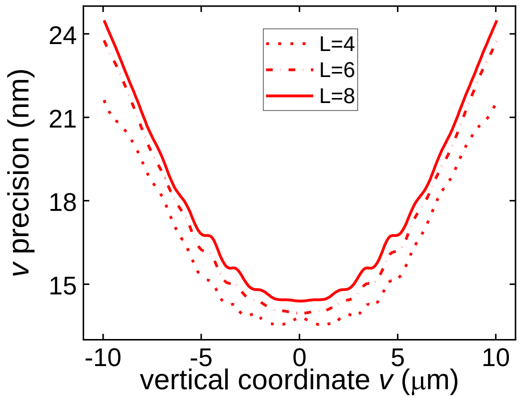

Supplementary Fig. 6. Influence of phase mask strength on the v precision, for $L = 4$, 6 and 8 respectively.

where (ρ, ϕ) are the normalized pupil coordinates, $A(\rho, \phi)$ is the pupil amplitude, $\psi(\rho, \phi)$ is the phase term introduced by the phase mask, n is the refractive index of the medium, λ is the wavelength of light, and NA is the numerical aperture of the objective. $D(\rho, z) = z\lambda^{-1}\sqrt{n^2 + \mathrm{NA}^2\rho^2}$ describes the defocus phase that occurs when the emitter is displaced by z from the focal plane [5]. We utilized the Fresnel-zone-based helical PSF design [6], and the phase mask optimization algorithm is described in Supplementary Fig. 5. It is modified from the Gerchberg–Saxton (GS) algorithm to account for PSF intensity distributions at various depths. The initial estimate of the phase mask is defined on circular zones in the Fourier plane as

$$\psi_0(\rho, \phi) = (2l - 1)\phi, \quad (\frac{l-1}{L})^\eta < \rho < (\frac{l}{L})^\eta, \quad l = 1, \cdots, L, \tag{15}$$

where L is the number of angular Fresnel zones with l referring to the l-th zone. η is a parameter that determines the helix peak confinement and the shape invariance during rotation [6]. A larger L leads to larger lobe spacing and slower angular rotation rate, and thus a larger operable depth range; theoretically, it yields a worse localization

precision in the axial direction (Supplementary Fig. 6). The initial amplitude function A_0 is the pupil aperture of the imaging system, and since a pure phase modulation is desired for high photon efficiency, the amplitude function keeps unchanged through out the iterative optimization.

For the n-th iteration, PSF images at various axial sampling points $(v_1 \cdots v_k \cdots v_K)$ are calculated from the current pupil function following Eq. (14). For each PSF image there will be two bright lobes, which are fitted to 2D Gaussian functions to locate each lobe. This yields the lobe coordinates (Z_k, H_k) and (Z'_k, H'_k) and their standard deviations σ and σ', based on which a 2D-two-peak weighting function w_k is constructed. Multiplying w_k to $I_{PSF}(z, h, v_k)$ boosts the two main lobes and suppress undesired light distributions in the background. Inverse Fourier transform of each amplitude PSF results in the light field in the Fourier plane, which divided by the defocus term leads to the updated phase modulations $\psi^{(n)}(\rho, \phi, v_k)$. Averaging through all the axial sampling points we get the phase mask function for the next iteration.

The iterative optimization converges and stops when the iteration number reaches a preset N or the average phase mask alteration between two successive iterations is bellow a preset $\Delta\Psi$. The optimization process smoothies the phase profile and reduces diffraction edges (i.e. phase jumps), which can be further smoothed by two-dimensional phase unwrapping.

The phase mask may be implemented in several ways, such as diffractive liquid-crystal SLM in a folded $4f$ configuration or refractive laser-written glass substrate in an inline configuration. When an SLM is implemented, the initial phase function ϕ_0 may be directly used with compromised performance. Besides, a linear phase ramp (or a blazed grating phase) may be added to the optimized phase mask function when using phase modulators with low fill factors to separate the unmodulated light from the modulated PSF. In the case of a glass substrate, smoothed surfaces are necessary to ease the fabrication process. In terms of optical efficiency: SLMs modulate one direction of polarization (50%) and yields a limited fill factor (around 90%), yielding an overall efficiency of around 45%. On the contrary, a glass phase mask can reach an efficiency of over 90% but at the expense of flexibility.

SUPPLEMENTARY NOTE 5. EXPERIMENTAL AND THEORETICAL COMPARISON BETWEEN HELICAL PSF AND THE DEFOCUSED AIRY DISK

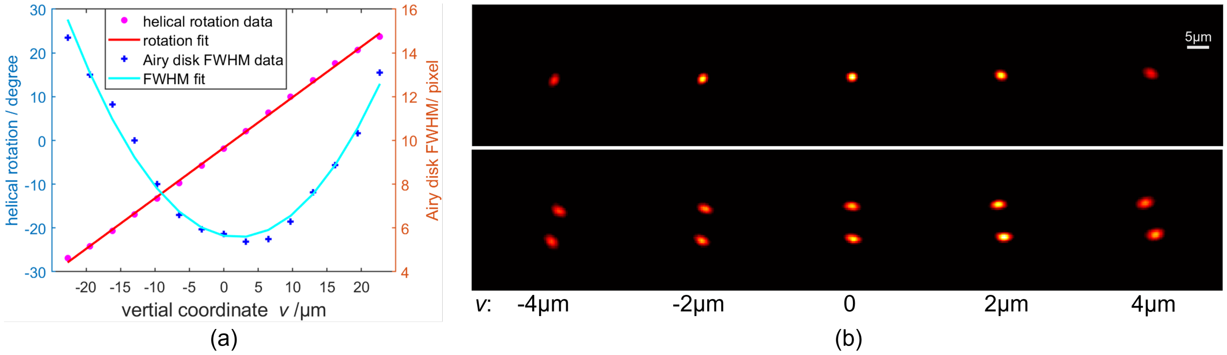

Supplementary Fig. 7. Experimental comparison between the conventional defocused Airy disk and the helical PSF. (a) Measured Airy disk diameter and helical PSF rotation angle as a function of the vertical coordinate. The points are experimental data and lines are fittings. (b) Stacked images of both defocused Airy disk (up) and helical PSFs (down) near the focal point.

An additional experimental comparison was performed between the helical PSF and the existing method of defocused Airy disk. We took image stacks of a single trapped ion by "pushing" it along the optical axis via the compensating electrodes. For each image in the stack, we measure the full-width-half maximum (i.e. $2\sqrt{2\ln 2}\sigma$) of the Airy disk and the rotation angle of the helical PSF, results can be seen in Supplementary Fig. 7(a). One can see that the helical PSF

keeps a consistent rotation rate, leading to a consistent depth sensitivity over the range of $-25\,\mu m$ to $25\,\mu m$; while the Airy disk diameter expands non-linearly and exhibits near-zero change rate near the focal plane. Corresponding images can be seen in Supplementary Fig. 7(b). This indicates that the Airy disk carries little information about the ion's axial position and yields no axial sensitivity near the focal plane, which agrees with the CRLB simulations in the Fig. 1f of main text.

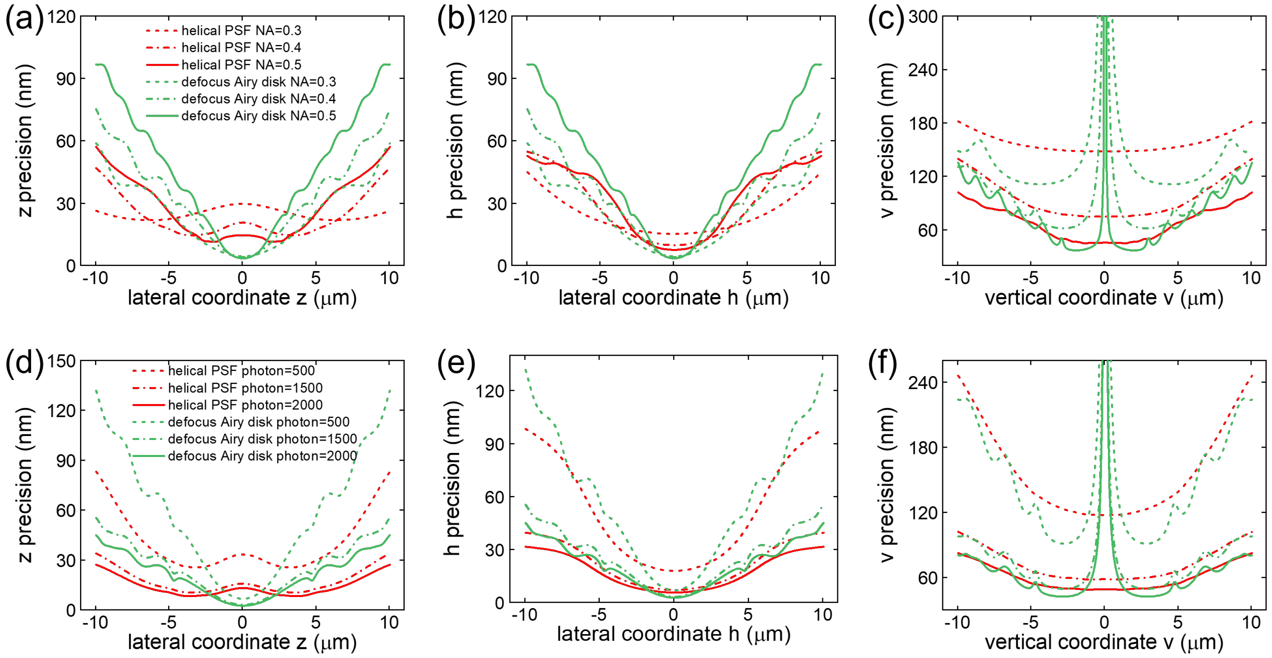

Supplementary Fig. 8. CRLB simulations for helical PSF and Airy disk method with various NAs and different levels of signal photon counts. (a-c) Theoretical precision for NA of 0.3, 0.4 and 0.5 respectively, simulated with total signal photon level of 1000 and average background of 2 photons per pixel. (d-f) Theoretical precision for signal photon levels of 500, 1500 and 2000, simulated with 0.4 NA and average background of 2 photons per pixel. (a) and (d) correspond to lateral direction z, (b) and (e) correspond to lateral direction h, (c) and (f) show vertical direction v, respectively.

The localization performance depends on the experimental conditions like numerical aperture and photon flux from the ion. Supplementary Fig. 8 shows the CRLBs in all three dimensions for the helical PSF and defocused Airy disk at various conditions. Supplementary Fig. 8(a-c) compare the theoretical precision for different NAs of 0.3, 0.4 and 0.5 respectively. As one can observe, localization precision in z, h, v for both methods improves with higher NA, however, at the expense of operable depth range. Of particular interest, the helical PSF shows a more consistent CRLB over the entire depth which compares favorably to the method of defocused Airy disk regardless of NA. Similarly, Supplementary Fig. 8(d-f) compare the theoretical precision for different signal photon levels of 500, 1500 and 2000 with NA fixed at 0.4. As expected, larger photon flux contributes to better localization performance for both methods and in all dimensions. Generally the helical PSF yields a more uniform localization performance in all three dimensions, and an advantageous CRLB in the axial direction (v) near the focal plane.

It's worth noting that the amount of Fisher information regarding the ion's 3D coordinates is calculated from variations in 3D light field, i.e. $\frac{\partial I_{PSF}}{\partial z}$, $\frac{\partial I_{PSF}}{\partial h}$ and $\frac{\partial I_{PSF}}{\partial v}$. An infinite CRLB in the v direction indicates that the light field of the Airy disk experiences no changes along the propagation direction at the focal plane, leading to its poor performance near the focus despite its high signal-to-noise ratio. If only the transverse position (h and z) is of concern, the conventional method of defocused Airy disk provides the best performance. Helical PSF becomes a better choice if the experiment requires information in 3D.

In addition, other types of engineered PSFs may be employed in 3D ion imaging potentially. For example, the Airy-beam-based PSFs [7, 8] offer the largest depth range with a compromised precision and the Tetrapod PSF is demonstrated in extremely high emitter density [9]. Several review articles can be referred to when choosing between different phase masks and corresponding engineered PSFs [10–12]. We look forward to the application of more PSF engineering techniques to be exploited in atomic-scale detection.

SUPPLEMENTARY REFERENCES

* These authors contributed equally
† pxchen@nudt.edu.cn
‡ zj1589233@126.com

[1] D. Leibfried, R. Blatt, C. Monroe, and D. Wineland, Quantum dynamics of single trapped ions, Rev. Mod. Phys. **75**, 281 (2003).

[2] S. Ejtemaee, *Dynamics of Trapped Ions Near the Linear-Zigzag Structural Phase Transition*, Ph.D. thesis, Simon Fraser University, Canada (2015).

[3] J. A. Kurvits, M. Jiang, and R. Zia, Comparative analysis of imaging configurations and objectives for fourier microscopy, J. Opt. Soc. Am. A **32**, 2082 (2015).

[4] C. W. McCutchen, Generalized aperture and the three-dimensional diffraction image, J. Opt. Soc. Am. **54**, 240 (1964).

[5] B. M. Hanser, M. G. L. Gustafsson, D. A. Agard, and J. W. Sedat, Phase retrieval for high-numerical-aperture optical systems, Opt. Lett. **28**, 801 (2003).

[6] R. Berlich and S. Stallinga, High-order-helix point spread functions for monocular three-dimensional imaging with superior aberration robustness, Optics express **26**, 4873—4891 (2018).

[7] S. Jia, J. C. Vaughan, and X. Zhuang, Isotropic three-dimensional super-resolution imaging with a self-bending point spread function, Nat. Photon. **8**, 302 (2014).

[8] Y. Zhou, P. Zammit, V. Zickus, J. M. Taylor, and A. R. Harvey, Twin-airy point-spread function for extended-volume particle localization, Phys. Rev. Lett. **124**, 198104 (2020).

[9] E. Nehme, D. Z. Freedman, R. Gordon, B. Ferdman, L. E. Weiss, O. Alalouf, T. Naor, R. Orange, T. Michaeli, and Y. Shechtman, DeepSTORM3D: dense 3D localization microscopy and PSF design by deep learning, Nature Methods **17**, 734 (2020).

[10] Y. Shechtman, Recent advances in point spread function engineering and related computational microscopy approaches: from one viewpoint, Biophysical Reviews **12**, 1303 (2020).

[11] Y. Zhou, M. Handley, G. Carles, and A. R. Harvey, Advances in 3D single particle localization microscopy, APL Photonics **4**, 060901 (2019).

[12] C. Manzo and M. F. García-Parajó, A review of progress in single particle tracking: from methods to biophysical insights, Reports on Progress in Physics **78**, 124601 (2015).
