# Supplementary material for: Tracking the extensive three-dimensional motion of single ions by an engineered point-spread function
Source: Nat Commun. 2024 Aug 1;15:6483. doi: 10.1038/s41467-024-49701-3 (PMC11294470; doi:10.1038/s41467-024-49701-3)
Supplement: Supplementary file 3 — Description of Additional Supplementary Files [file 41467_2024_49701_MOESM3_ESM.pdf]

Description of Additional Supplementary Files

File Name: Supplementary Movie 1

Description: Tracking the 3D vibration of a single Ca ion

File Name: Supplementary Movie 2

Description: Monitoring the zigzag structural phase transition of an ion chain
